# Monocytosis associated with the growth of transplanted syngeneic rat sarcomata differing in immunogenicity.

**DOI:** 10.1038/bjc.1976.116

**Published:** 1976-07

**Authors:** S. A. Eccles, G. Bandlow, P. Alexander

## Abstract

The effect of the growth of two syngeneic transplanted sarcomata of widely differing biological properties on the number of monocytes in the blood of rats was measured (1) by binding of a specific antimacrophage serum to leucocytes, and (2) by sedimenting in a density gradient rosettes between mononuclear cells and antibody-coated sheep red cells under conditions in which B-cells are not brought down. For the 4 syngeneic sarcomata studied there was a progressive increase in the number of monocytes with tumour growth and the values returned to normal a few days after their surgical removal. The extent of monocytosis was related to the immunogenicity of the tumour and was most pronounced for the HSBPA sarcoma, which is highly immunogenic, has a low rate of spontaneous metastasis and contains many macrophages, and least for the MC-3 sarcoma which is essentially non-immunogenic, invariably gives rise to distant metastases and contains only about 8% macrophages. The growth of sarcomata had previously been found to reduce the number of monocytes which enter inflammatory lesions, both non-specific and due to a delayed hypersensitivity reaction. This "anti-inflammatory" action of sarcomata which is related to their immunogenicity cannot be ascribed to the preferential uptake of monocytes by the tumours and it is concluded that the monocytes in the blood of tumour-bearers, though increased in number, are modified so that they do not enter sites of inflammation.


Br. J. Cancer (1976) 34, 20

### MONOCYTOSIS ASSOCIATED WITH THE GROWTH OF TRANSPLANTED SYNGENEIC RAT SARCOMATA DIFFERING IN IMMUNOGENICITY

S. A. ECCLES, G. BANDLOW AND P. ALEXANDER

*From the Division of Tumour Immunology, Chester Beatty Research Institute, Sutton, Surrey, England*

Received 4 February 1976 Accepted 11 March 1976

**Summary.—The effect of the growth of two syngeneic transplanted sarcomata of widely differing biological properties on the number of monocytes in the blood of rats was measured (1) by binding of a specific antimacrophage serum to leucocytes, and (2) by sedimenting in a density gradient rosettes between mononuclear cells and antibody-coated sheep red cells under conditions in which B-cells are not brought down. For the 4 syngeneic sarcomata studied there was a progressive increase in the number of monocytes with tumour growth and the values returned to normal a few days after their surgical removal. The extent of monocytosis was related to the immunogenicity of the tumour and was most pronounced for the HSBPA sarcoma, which is highly immunogenic, has a low rate of spontaneous metastasis and contains many macrophages, and least for the MC-3 sarcoma which is essentially non-immunogenic, invariably gives rise to distant metastases and contains only about 8% macrophages. The growth of sarcomata had previously been found to reduce the number of monocytes which enter inflammatory lesions, both non-specific and due to a delayed hypersensitivity reaction. This "anti-inflammatory" action of sarcomata which is related to their immunogenicity cannot be ascribed to the preferential uptake of monocytes by the tumours and it is concluded that the monocytes in the blood of tumour-bearers, though increased in number, are modified so that they do not enter sites of inflammation.**

In a previous study (Eccles and Alexander, 1974*a*) syngeneic transplanted sarcomata were found to vary widely in the extent to which they reduced the capacity of rats to mount non-specific inflammatory reactions, as well as delayed hypersensitivity responses to antigens unrelated to the tumour. The former was measured by counting the number of macrophages recoverable from the peritoneal cavity 4 days after stimulation with oyster glycogen (in normal rats this produced an increase from 2 to $14 \times 10^6$ macrophages per rat). The effect on delayed hypersensitivity was assessed by measuring the degree of swelling of foot pads following injection of either sheep red blood cells (SRBC) or PPD into rats which had been previously sensitized with SRBC or BCG respectively.

A number of tests showed that this "anti-inflammatory" effect of tumour growth was due to a failure of monocytes to extravasate into the sites of inflammation or antigen challenge and was not caused by a reduction in the number of allergized lymphocytes. The intensity of both types of reaction decreased progressively as the tumours grew and returned to normal within 6 days of tumour excision. For a given size of tumour, there was a correlation between the degree of suppression and the macrophage content of the tumour (which for the different sarcomata studied ranged from 3 to 58% of the total cells). Since

Evans (1972) had shown that the macrophages in the tumour are of host origin, and as they do not divide *in situ* they must all be derived from blood monocytes. This suggested that the failure of monocytes to reach sites of inflammation in tumour-bearing rats might be caused by sequestration of the circulating monocytes within the tumour. A corollary of this hypothesis is that the number of circulating monocytes in the blood should fall in parallel with the observed " anti-inflammatory " effects.

Monocytes, unlike their mature counterparts—macrophages—cannot always be reliably identified using morphological criteria alone, and the immature forms which tended to appear in the blood of tumour-bearing animals were particularly difficult to distinguish from other early cell types. For this reason two alternative methods, which depend on functional properties were devised to identify blood monocytes:

(1) Membrane fluorescence using a specific rabbit anti-rat macrophage serum (RAMS). (2) Separation by density sedimentation of rosettes formed between monocytes and antibody-coated sheep red cells (EA rosettes). These tests showed that growth of sarcomata induced a marked monocytosis and the " anti-inflammatory " action cannot, therefore, be ascribed to the preferential attraction of monocytes to the tumour leading to a depletion of monocytes elsewhere. Consequently, the mechanism whereby monocytes are able to enter tumours but have a reduced capacity to enter sites of inflammation or antigen challenge, has yet to be explained.

### MATERIALS AND METHODS

*Transplantation of tumours.*—The chemically-induced sarcomata were transplanted by mechanically prepared cell suspensions into the legs of syngeneic male 10-week-old " hooded " rats. The average macrophage contents of the HSBPA, HSN, MC-1 and MC-3 sarcomata were respectively 54%, 40%, 38% and 8%. Their biological properties have been described previously (Eccles and Alexander, 1974*a*, *b*).

*Preparation of blood mononuclear cells.*—Heparinized blood was obtained from the jugular vein of anaesthetized animals and a total white blood cell count was made. The whole blood was then layered on to an equal volume of Ficoll–Triosil (density 1·077 gm/ml) at room temperature. The tubes were spun at 200 *g* for 10 min, then 600 *g* for 15 min. The upper cell layer containing mononuclear cells was taken, washed twice with Medium 199, and the cells counted and expressed per ml of whole blood. The monocyte content of this fraction was then assayed by the following methods and expressed as number of cells/ml of blood.

*Production of anti-macrophage serum (RAMS).*—Rat peritoneal cells were harvested 3 days after the i.p. injection of 4 mg oyster glycogen and plated into 3-cm Petri dishes. The cells were incubated overnight in serum-free medium at 37 °C, which allowed strong adherence of the mononuclear phagocytes to the plastic. The non-adherent cells were washed off and discarded, and the adherent cells removed with a rubber policeman, and used for immunization of a rabbit. The first immunization consisted of $10^7$ macrophages emulsified with complete Freund's adjuvant injected s.c., and $10^6$ macrophages injected i.v. Three subsequent immunizations consisting of $10^7$ macrophages in incomplete Freund's adjuvant injected s.c. were given, and the rabbit bled 7–10 days after the last dose. The serum was absorbed overnight at 4 °C against equal volumes of packed cells of each of the following types: (a) rat red blood cells, (b) thoracic duct lymphocytes, and (c) cultured syngeneic tumour cells. The serum was then spun for 6 h at 100,000 *g* to remove immune complexes, and tested with rat or rabbit serum as a complement source for lysis of various cell types. No cytotoxic activity was detected against thoracic duct lymphocytes, polymorphs or tumour cells. The serum (at a dilution of 1 : 100) plus complement caused complete lysis of all rat macrophages in a monolayer prepared from peritoneal exudate cells.

*Assay of monocytes by immune fluorescence with RAMS.*—$3 \times 10^6$ blood mononuclear cells were placed in siliconized tubes, and 0·5 ml of RAMS at a final dilution of 1 : 100 was added, and the mixture incubated at 4°C for $1\frac{1}{2}$ h. The cells were then washed twice, goat anti-rabbit serum conjugated with fluorescein (Burroughs Wellcome) added at a dilution of 1 : 10, and incubated at 4°C for 1 h. The cells were then washed 3 times in Medium 199, spun down, and resuspended in 2 drops of buffered glycerol. One drop was placed on a slide and mounted under a coverslip. These preparations were examined with a Leitz u.v. microscope and positively stained cells counted. Cells of the monocyte/macrophage series stained positively with this preparation of RAMS under the conditions described. No staining was found with thoracic duct lymph cells, cultured sarcoma cells, leukaemia cells (*i.e.* the SAL myeloid rat leukaemia or the HRL rat lymphatic leukaemia described by Wrathmell (1976)) or cultured normal rat embryo fibroblasts.

*Preparation of antibody-coated sheep red cells (EA cells)*.—Sheep red blood cells (SRBC) were washed 3 times and maintained in Fischer's medium at a concentration of 4% packed volume. Rat anti-sheep red blood cell serum was obtained by immunizing rats i.v. 4 times with washed SRBC ($2 \times 10^8$ SRBC followed 3, 6 and 13 days later by $10^8$ SRBC; the serum was taken on Day 20). The blood was allowed to clot, the serum separated and heat-inactivated at 56°C for 30 min. It was added to the SRBC at a final dilution of 1 : 100, incubated for 1 h at 37°C, after which the cells were washed carefully to remove unbound antibody. The cells were then ready for the EA rosette test.

*Assay of monocytes by density separation of rosettes formed with EA cells*.—Mononuclear cells obtained from blood were washed 3 times with Medium 199 to remove traces of serum containing complement activity. The viability of these cells was always greater than 95%. The mononuclear cells were made up to a concentration of $3 \times 10^6$ cells/ml, and added to an equal volume of EA to give a final concentration of 2% of packed red cells. These concentrations are quite critical, and had been worked out in pilot experiments to achieve a separation of B-lymphocytes from monocytes. The mononuclear–EA cell mixture was then immediately layered on to an equal volume of Ficoll–Triosil, and spun again as before at room temperature. Two cell layers were formed, both were removed, washed and examined further. The cell pellet consisted of SRBC, together with rosetting mononuclear cells, which could be recovered intact and counted. Less than 2% of the cells in the pellet were free (*i.e.* unrosetted) viable mononuclear cells, although some dead (*i.e.* trypan blue +ve) cells were present. The upper layer consisted of non-rosetted mononuclear cells.

The cells in the pellet were resuspended in Fischer's medium and the number of mononuclear cells present were counted. More than 90% of these were shown (see Results section) to be monocytes. Although both B-lymphocytes and monocytes can form EA rosettes, under the particular conditions employed very few rat B-lymphocytes were recovered.

### RESULTS

### *Validity of the procedures used for quantitative assay of blood monocytes*

The tests described in the Materials and Methods section show that the RAMS is specific for macrophages. It stained on average $1{\cdot}2 \times 10^5$ mononuclear cells/ml of blood from normal rats and this value corresponded closely to the number of macrophages which could be cultured from this volume of blood. Removal of cells which had bound carbonyl iron with a magnet reduced the number of cells staining to less than one-fifth. We conclude that carefully absorbed RAMS recognizes a surface marker which is present on all of the cells belonging to the mononuclear phagocyte series except perhaps monoblasts (i.e. promonocytes, monocytes and macrophages).

At first sight it is surprising that the procedure of sedimenting mononuclear cells which have formed EA rosettes gives rise to a preparation that contains so few B-lymphocytes, since these also have Fc receptors, and Parish and Hayward (1974) found that rat B-cells appeared as EA rosettes in the pellet of a Ficoll–Triosil gradient. Parish and Hayward, however, used a much higher EA : mononuclear cell ratio (*i.e.* 10% EA as opposed to 2% in the procedure used by us), a higher Ficoll–Triosil density (1·09 as opposed to 1·077 g/ml) higher *g* forces in the separation, and an incubation period of EA and mononuclear cells prior to separation, while in our procedure separation begins immediately after mixing. The two techniques were compared using rat thoracic duct lymph cells which contain B and T lymphocytes but no monocytes. By the Parish and Hayward method, 15% of the thoracic duct cells were spun down as EA rosettes, whereas <2% were pelleted in the procedure used by us.

Table I summarizes the evidence that at least 90% of the cells we obtained in the pellet of blood mononuclear cells were monocytes. If the cells which had sedimented as EA rosettes were incubated in suspension in Fisher's medium for 30 min, between 60 and 70% of them could immediately be seen to be phagocytes by the presence within them of intact red cells. This, however, underestimates the number of phagocytic cells, since some of these will have digested the red cells so that they are no longer visible, but such cells still contain haemoglobin, which can be visualized by appropriate staining, giving values of 90–95% phagocytes.

The red cells in the pellet can be readily removed by hypotonic lysis and all of the mononuclear cells recovered. On culturing for 15 min at 37°C between 50 to 70% of these cells adhered to the bottom of plastic culture wells. However, after 24 h of culture more than 90% of the cells had adhered and spread; all of these phagocytosed EA and also on morphological criteria were obviously macrophages. Cells sedimented as EA rosettes from the blood of rats immunized with SRBC were tested for antibody production by the modified Jerne plaque technique (Cunningham, 1965) but no positive cells were observed. The RAMS also identifies at least 90% of the cells in the pellet as mononuclear phagocytic cells by immunofluorescence and by causing complement-dependent lysis.

From these experiments we concluded that the procedure described in this paper for sedimenting EA rosettes formed with mononuclear cells from rat blood results in a preparation which contains less than 5% B-cells. The evidence that essentially all of the monocytes were recovered is that in the upper layer less than 1% interacted with RAMS, adhered to glass on culture for 24 h, or phagocytosed EA cells. *In toto* these data show that the sedimentation technique described, which relies on qualitative differences between EA rosettes of B-cells and monocytes, is capable of providing a method for enumerating the monocytes in the blood of rats. Whether a similar procedure is applicable to all other species remains to be established, although preliminary human data is promising.

### *Monocytosis associated with tumour growth*

The two transplanted syngeneic sarcomata, HSBPA and MC-3, grow at approximately equal rates in the hind limbs of rats following transplantation. The monocyte count in normal rats showed periodic variations and ranged

TABLE I.—*Percentage of Monocytes among Nucleated Cells Sedimenting with Antibody-coated Sheep Red Blood Cells (EA) as Revealed by Different Functional Tests*

| Assay | % Positive cells | Function |
|---|---|---|
| Immune phagocytosis (cells containing intact SRBC) | 60–70 | Phagocytosis |
| Intracellular haemoglobin staining (Benzidine) | 90 | |
| Glass adherence (after 15 min at 37°C) | 50–70 | Adherence |
| Transformation to macrophages after overnight incubation | >90 | |
| RAMS—fluorescent staining | 90 | Binding of specific antisera |
| (RAMS + Complement)-lysis | 90 | |
| Plaque formation by haemolysis (Jerne technique) | 0 | Antibody production |

FIG.—Number of peripheral blood monocytes in tumour-bearing male hooded rats assayed at intervals during tumour growth by EA rosette method. ——▲—— HSBPA-tumour-bearing rats. ——●—— MC-3-tumour-bearing rats. – – –△– – – Post-excision HSBPA tumour. – – –○– – – Post-excision MC-3 tumour. Each point represents the mean value from 5 animals, and the vertical bars show the range for the group. The two tumours grew at approximately equal rates, attaining average weights of 4·2 g on Day 7, 10·4 g on Day 10, 15 g on Day 14 and 22·5 g on Day 18.

from 0·5 to 1·5 $\times$ $10^5$/ml of blood. At the time when this experiment was performed, the number found in the blood of stock rats was in the lower range of 0·5 to 1·0 $\times$ $10^5$/ml. The figure shows that both sarcomata induced a progressive monocytosis but this effect was greater with the HSBPA, in which the number of monocytes had increased approximately 8-fold 14 days after transplant when the tumours weighed an average of 15 g.

Two other tumours, HSN and MC-1, with immunogenicities and metastatic capacities between those of HSBPA and MC-3 (Eccles and Alexander, 1974*b*), were also tested for their effects on monocyte levels at 10 days after inoculation, and the values obtained were 0·52 $\times$ $10^6$ and 0·46 $\times$ $10^6$/ml respectively, which fall between the figures obtained for HSBPA and MC-3 at this stage of growth.

Following surgical removal of the tumour by amputation of the leg, the number of blood monocytes returned to the normal range within 5 days. In control rats, amputation of the leg did not cause the number of monocytes to fluctuate outside the range normally found (*i.e.* 0·5–1·5 $\times$ $10^5$/ml).

Although the EA rosette sedimentation method had been used for the sequential analysis of blood monocytes during tumour growth, it was felt that the values obtained should be checked using an entirely independent assay. For this reason, selected blood samples were also assayed for blood monocytes using the fluorescent RAMS method. The results are shown in Table II, and it can be seen that both methods give closely comparable results.

Infection, particularly with intracellular organisms (Volkman and Collins, 1974), is known to induce moderate monocytosis in rodents. While infection introduced with the transplant cannot be definitely excluded as the cause for the monocytosis seen in the tumour-

TABLE II.—*Number of Monocytes/ml of Blood*

| Rats | Assay method* RAMS-fluorescence | EA rosette formation in Ficoll–Triosil |
|---|---|---|
| HSBPA-tumour-bearing (Day 15) | $6 \cdot 3\ (\pm 0 \cdot 8) \times 10^5$ | $7 \cdot 8\ (\pm 0 \cdot 3) \times 10^5$ |
| MC-3-tumour-bearing (Day 16) | $2 \cdot 4\ (\pm 0 \cdot 6) \times 10^5$ | $3\ \ (\pm 0 \cdot 4) \times 10^5$ |
| Normal age-matched controls | $1 \cdot 2\ (\pm 0 \cdot 3) \times 10^5$ | $0 \cdot 9\ (\pm 0 \cdot 2) \times 10^5$ |

* The figures given represent the mean values obtained in groups of 5 rats with the standard errors following in brackets.

bearing rats, this seems improbable for the following reasons: (1) On culture of the tumours by the microbiological service of the M.R.C. Laboratory Animal Centre, no bacteria or mycoplasma could be detected. (2) No viruses were detected in sections examined in the electron microscope. (3) Inoculation of cell-free homogenates did not produce a monocytosis. (4) Similar findings were made in 3 separate experiments carried out over a period of 12 months. In these separate experiments the tumours were derived from a different stock of cells—it is our practice to transplant a tumour only 10–15 times before starting a new series from a store of deep-frozen cells. If infection were responsible, then the agent can only be propagated within a growing tumour.

### DISCUSSION

Our observation that the growth of rat sarcomata is accompanied by monocytosis is in agreement with earlier findings that transplanted tumours can stimulate the RE system and increase the number of granulocyte and macrophage precursors in bone marrow (Lappat and Cawein, 1964; Hibberd and Metcalf, 1971; Baum and Fisher, 1972). The experiments reported were carried out in the hope of elucidating the reasons for the differences in anti-inflammatory action of different sarcomata (Eccles and Alexander, 1974*a*). The magnitude of this effect was directly correlated with the macrophage content of the tumours but the simple explanation that the suppression of monocyte movement was caused by the successful competition by the tumour for the available monocytes appears now to be excluded. Rats with large HSBPA tumours have up to 8 times as many monocytes in the blood as normal rats, but these monocytes do not reach sites of inflammation. Yet clearly these same monocytes gain access to tumours and it would therefore appear that entry of monocytes into tumours is a different process from entry of monocytes into sites of inflammation or delayed hypersensitivity.

A crude estimation shows that in rats with an HSBPA sarcoma growing at the rate of 1 g/day, approximately $10^8$ monocytes/day must enter the tumour, since the percentage of macrophages remains more or less constant. Normal rats have a turnover rate of around $3{\cdot}6 \times 10^6$ monocytes/day (Whitelaw, 1966) and therefore to supply the monocytes entering the tumour, their output has to be vastly increased. The bone marrow is clearly capable of responding to this demand since the rat is not monocytopaenic but on the contrary the number of blood monocytes is raised by a factor of up to 8—although this does plateau, implying an upper limit of production. The stimulus may be provided by a factor released from the tumour which acts on the bone marrow or be associated with the immune stimulus provided by the tumour-specific antigens.

The possibility that the difference in the degree of monocytosis induced by the tumours studied here may be related to the immune response of the host appears to find support from a previous study (Eccles and Alexander, 1974*b*) in which correlations had been observed between the biological properties of different sarcomata and their macrophage content. The two tumours investigated most extensively in the present study represent the extreme ends of the spectrum. The HSBPA contains around 54% macrophages, and following surgical excision after 14 days of growth, only 10% of the rats develop distant metastases. The MC-3 sarcoma has 8% macrophages, and 100% of the rats die within 6 months from distant metastases following removal of the local sarcoma. Immunogenicity—defined by the number of tumour cells which an immunized rat is capable of rejecting—parallels the macrophage content and is inversely related to metastasis, *i.e.* rats immunized with HSBPA are capable of rejecting more than $10^7$ intramuscular (i.m.) HSBPA cells, whereas in rats immunized with MC-3 no resistance to a subsequent i.m. challenge could be detected (Eccles and Alexander, 1974*b*). However, absense of immunogenicity as defined by graft rejection does not necessarily imply that there is no host reaction to antigens on the membrane of the tumour, and the lymphoid cells in the node draining sarcomata of widely differing immunogenicities displayed comparable tumour-specific cytotoxicity *in vitro* (Currie and Alexander, 1974). The finding that the MC-3 evokes a specific host response and yet is not "immunogenic" has been attributed (Alexander, 1974) to its ability to escape destruction by cell-mediated immunity by shedding tumour antigens, which pre-empt the attacking cells. If the difference in the extent of the monocytosis produced by the tumours has an immunological basis, then the mechanism must be complex.

Whatever the basis of the tumour-induced monocytosis, there remains the puzzle as to why these monocytes fail to enter sites of inflammation. This also may be linked to immunity and in particular to the presence of immune complexes containing shed tumour-specific antigens in the tissue fluids of tumour-bearing rats, the existence of which has been indicated in earlier investigations using these sarcomata (Thomson, Eccles and Alexander, 1973). We are testing the hypothesis that binding of immune complexes to the Fc receptors of monocytes interferes either directly or by steric hindrance with the membrane recognition of sites involved in extravasation and/or chemotaxis, the processes needed for monocytes to enter sites of inflammation and hypersensitivity reactions.

We wish to thank Dr C. L. Gauci for help and advice in the use of the anti-macrophage serum. Dr G. Bandlow was supported by a Royal Society Anglo–German exchange Fellowship. An M.R.C. Programme Grant provided support for this investigation.

REFERENCES

Alexander, P. (1974) Escape from Immune Destruction by the Host through Shedding of Surface Antigens: Is this a Characteristic Shared by Malignant and Embryonic Cells? *Cancer Res.*, **34**, 2077.

Baum, M. & Fisher, B. (1972) Macrophage Production by the Bone Marrow of Tumor-bearing Mice. *Cancer Res.*, **32**, 2813.

Cunningham, A. J. (1965) A Method of Increased Sensitivity for Detecting Single Antibody-forming Cells. *Nature, Lond.*, **207**, 1106.

Currie, G. A. & Alexander, P. (1974) Spontaneous Shedding of TSTA by Viable Sarcoma Cells: its Possible Role in Facilitating Metastatic Spread. *Br. J. Cancer*, **29**, 72.

Eccles, S. A. & Alexander, P. (1974*a*) Sequestration of Macrophages in Growing Tumours and its Effect on the Immunological Capacity of the Host. *Br. J. Cancer*, **30**, 42.

Eccles, S. A. & Alexander, P. (1974*b*) Macrophage Content of Tumours in Relation to Metastatic Spread and Host Immune Reaction. *Nature, Lond.*, **250**, 667.

Evans, R. (1972) Macrophages in Syngeneic Animal Tumours. *Transplantation*, **14**, 468.

Hibberd, A. D. & Metcalf, D. (1971) Proliferation of Macrophage and Granulocyte Precursors in Response to Primary and Transplanted Tumors. *Israel J. med. Sci.*, **7**, 202.

Lappat, E. J. & Cawein, M. (1964) A Study of the Leukemoid Response to Transplantable A-280 Tumor in Mice. *Cancer Res.*, **24**, 302.

Parish, C. R. & Hayward, J. A. (1974) The Lymphocyte Surface. II. Separation of Fc Receptor, C'3 Receptor and Surface Immunoglobulin-bearing Lymphocytes. *Proc. R. Soc.*, B, **187**, 65.

THOMSON, D. M. P., ECCLES, S. & ALEXANDER, P. (1973) Antibodies and Soluble Tumour-specific Antigens in Blood and Lymph of Rats with Chemically Induced Sarcomata. *Br. J. Cancer*, **28**, 6.

VOLKMAN, A. & COLLINS, F. M. (1974) The Cytokinetics of Monocytosis in Acute *Salmonella* Infection in the Rat. *J. exp. Med.*, **139**, 264.

WHITELAW, D. M. (1966) The Intravascular Lifespan of Monocytes. *Blood*, **28**, 455.

WRATHMELL, A. B. (1976) The Growth Patterns of Two Transplantable Acute Leukaemias of Spontaneous Origin in Rats. *Br. J. Cancer*, **33**, 172.


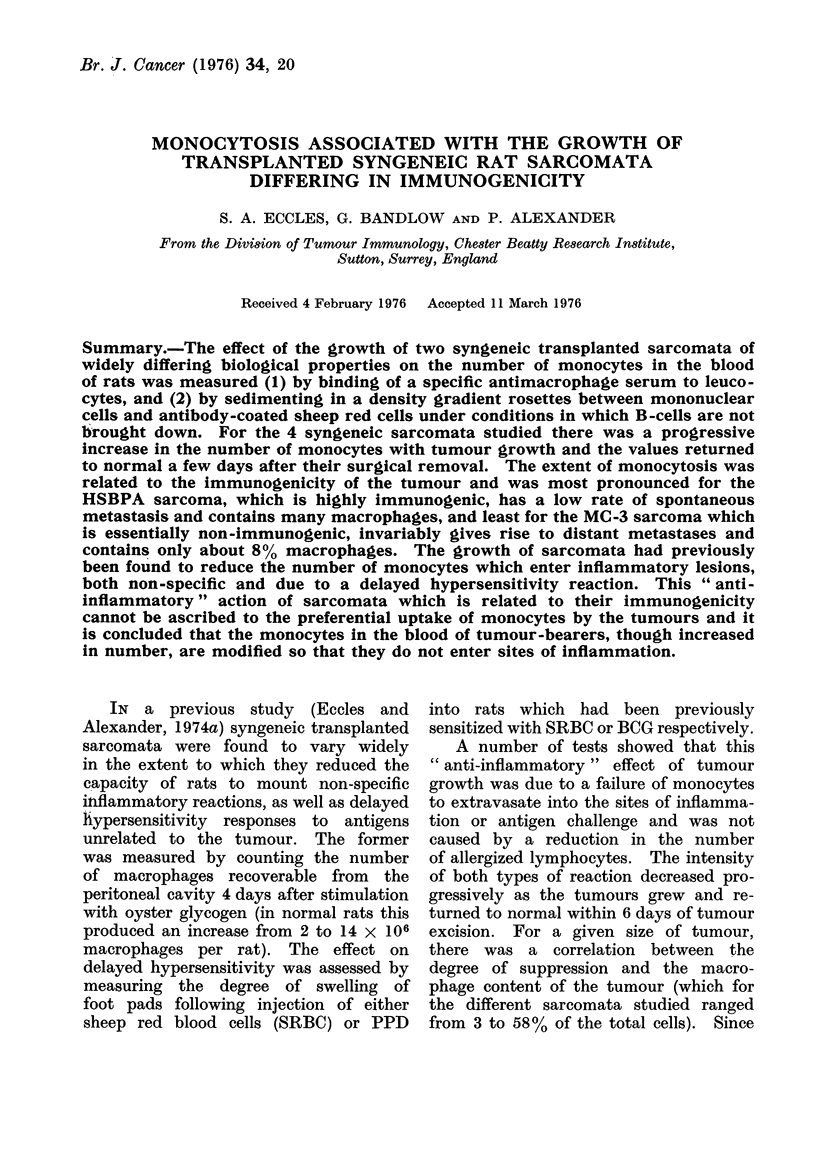

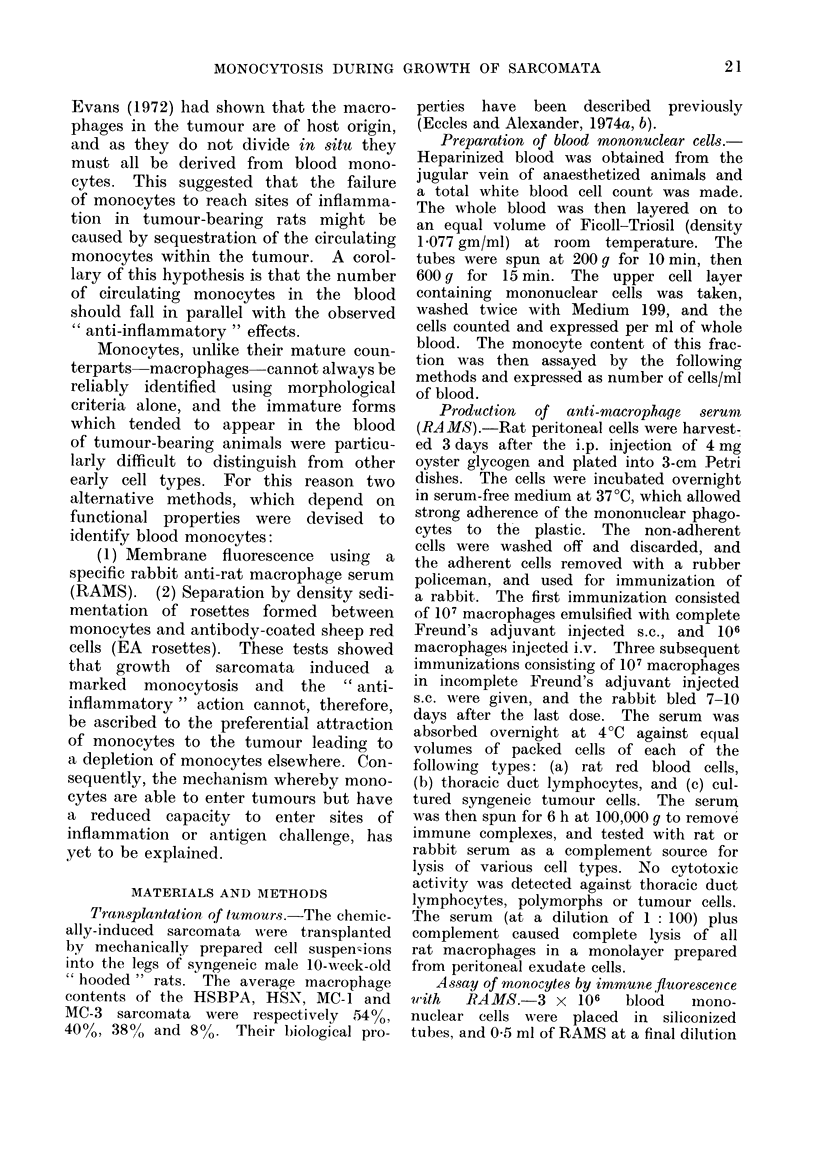

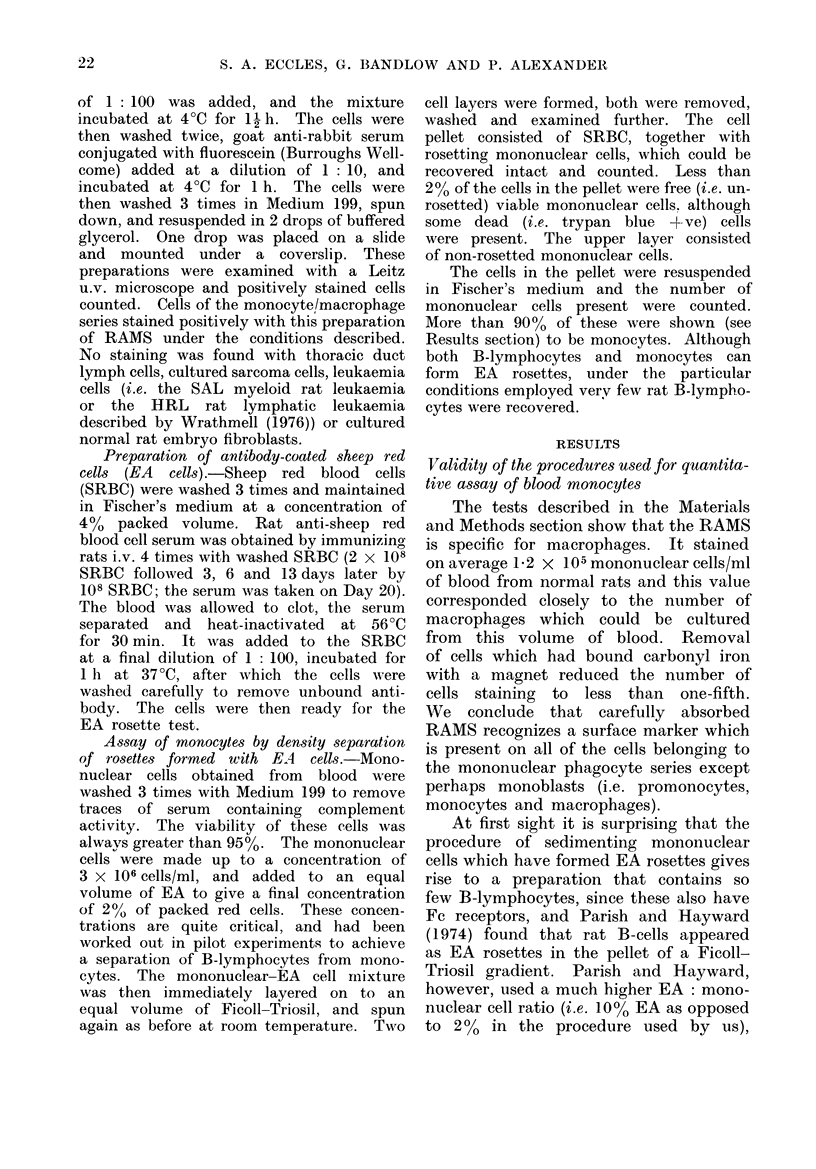

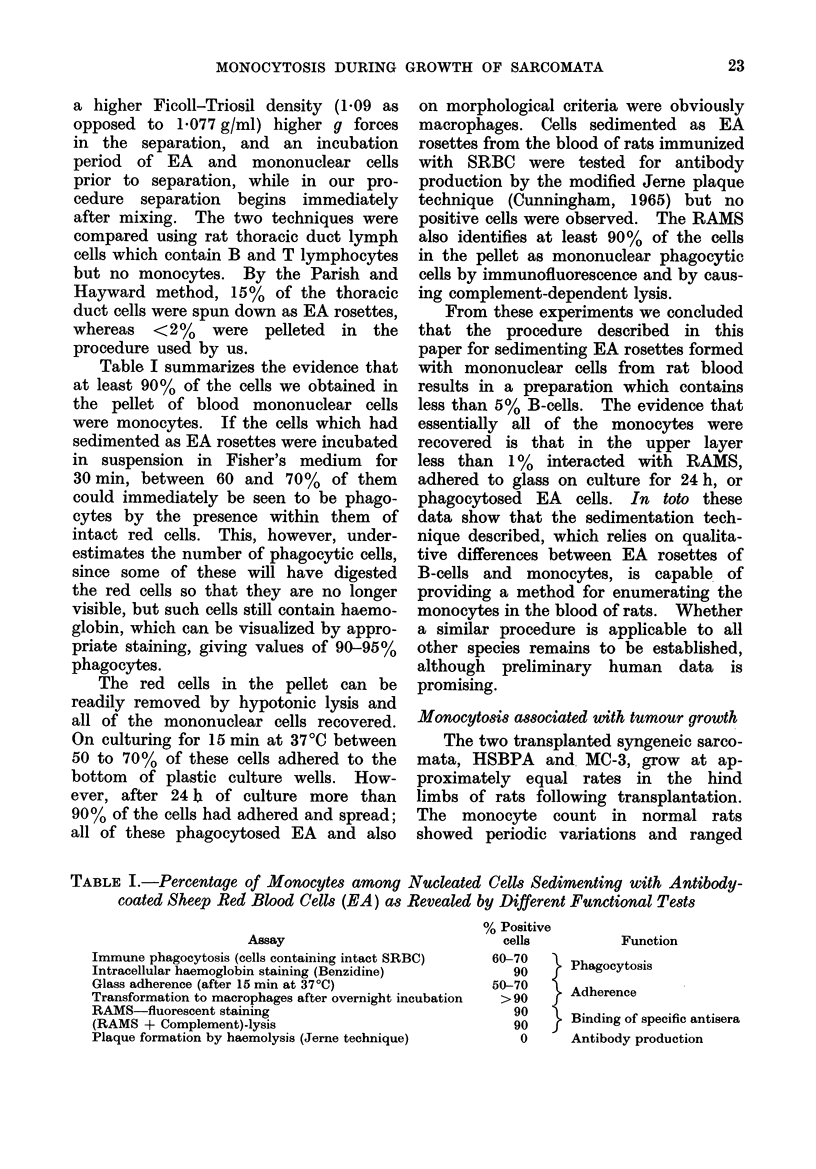

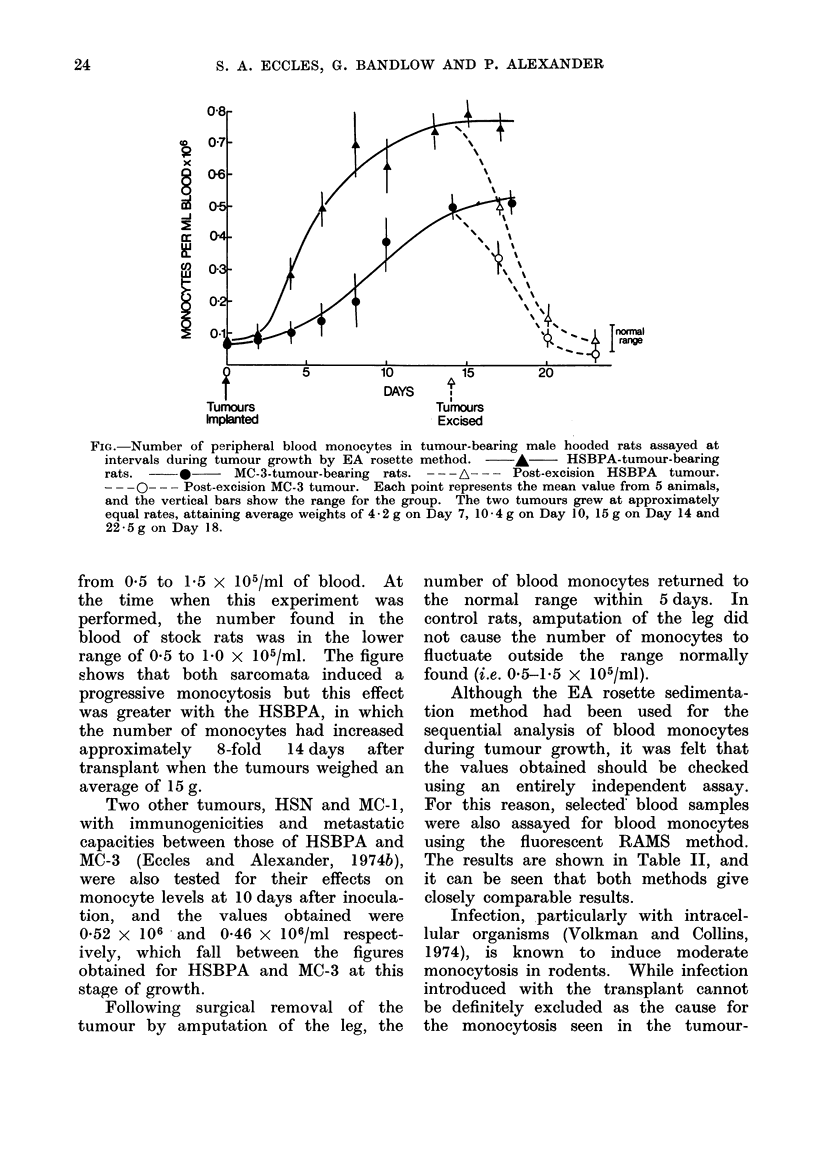

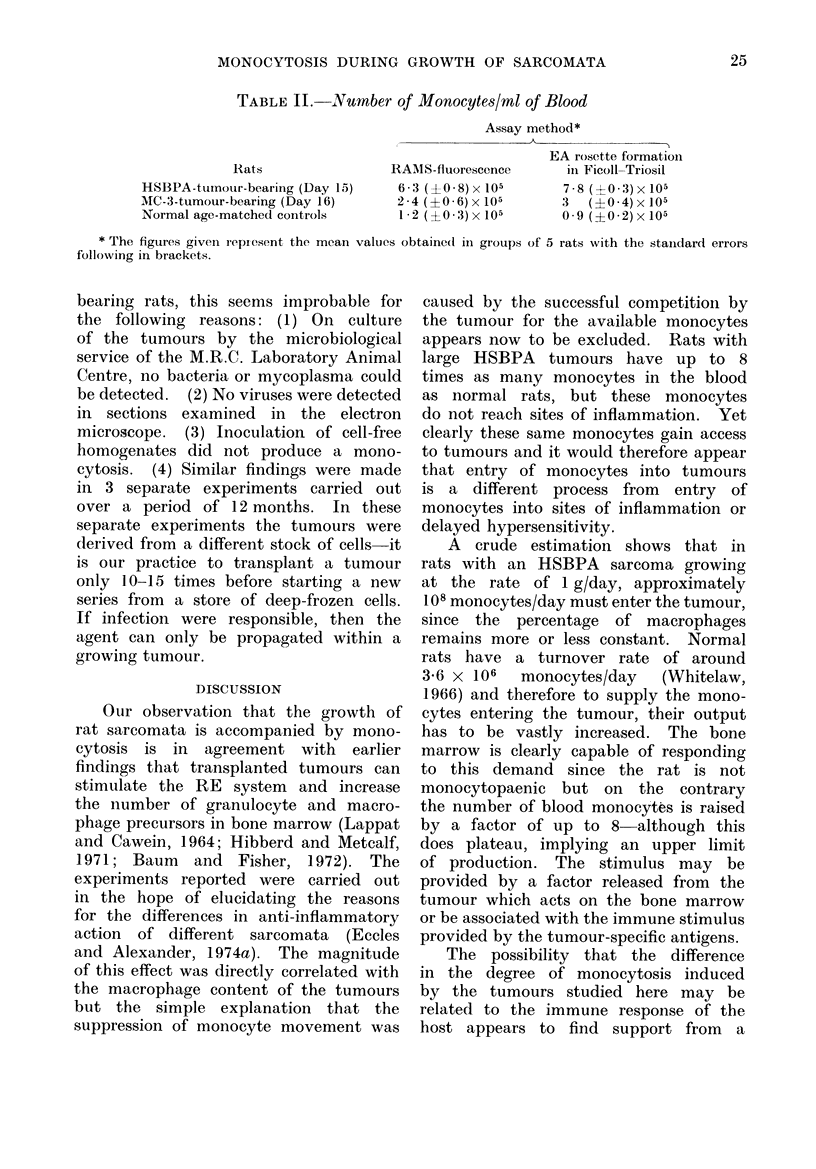

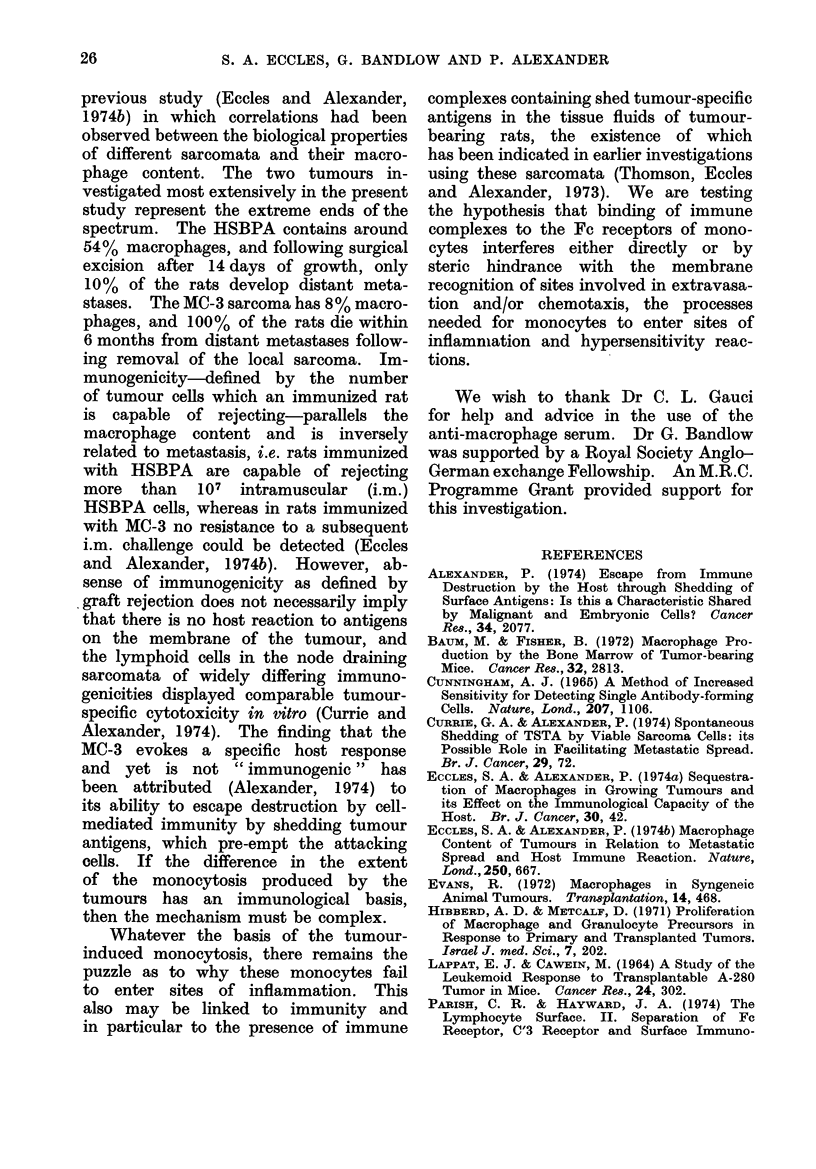

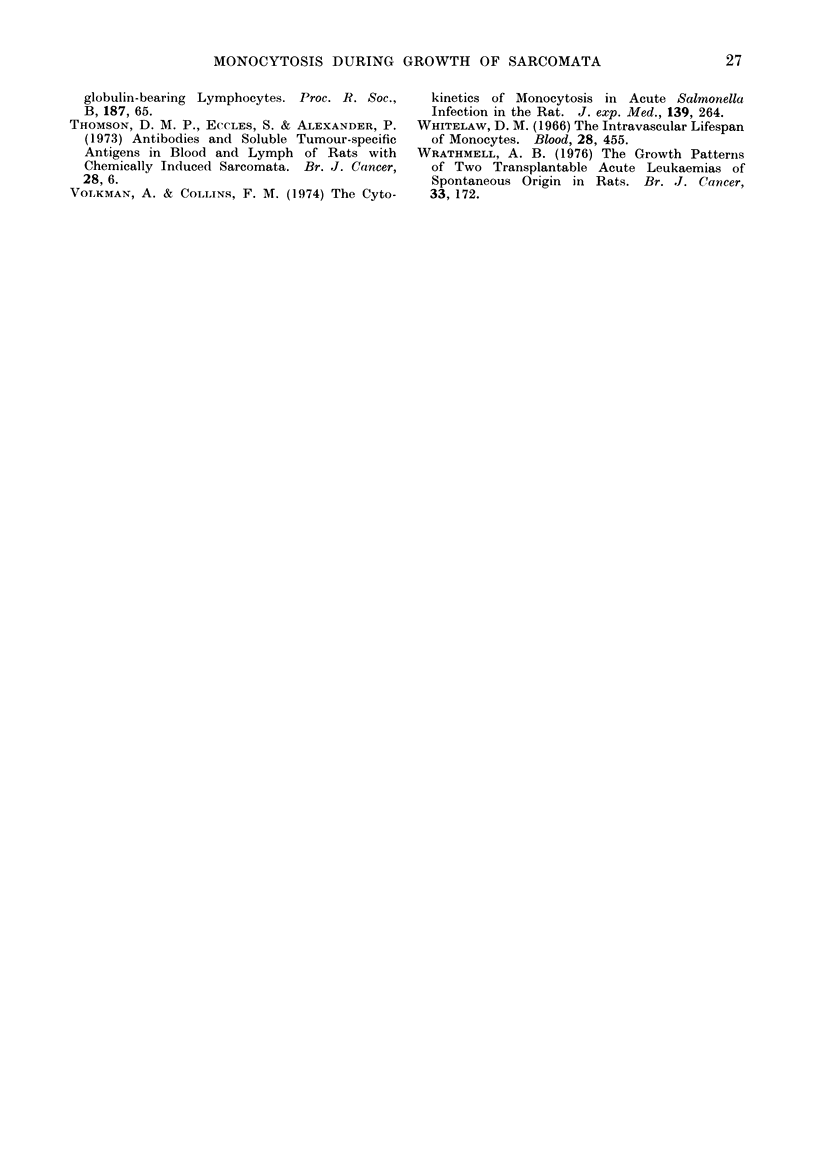


## References

[OCR_00724] Alexander P. (1974). Proceedings: Escape from immune destruction by the host through shedding of surface antigens: is this a characteristic shared by malignant and embryonic cells?. Cancer Res.

[OCR_00731] Baum M., Fisher B. (1972). Macrophage production by the bone marrow of tumor-bearing mice.. Cancer Res.

[OCR_00736] Cunningham A. J. (1965). A method of increased sensitivity for detecting single antibody-forming cells.. Nature.

[OCR_00741] Currie G. A., Alexander P. (1974). Spontaneous shedding of TSTA by viable sarcoma cells: its possible role in facilitating metastatic spread.. Br J Cancer.

[OCR_00753] Eccles S. A., Alexander P. (1974). Macrophage content of tumours in relation to metastatic spread and host immune reaction.. Nature.

[OCR_00747] Eccles S. A., Alexander P. (1974). Sequestration of macrophages in growing tumours and its effect on the immunological capacity of the host.. Br J Cancer.

[OCR_00759] Evans R. (1972). Macrophages in syngeneic animal tumours.. Transplantation.

[OCR_00763] Hibberd A. D., Metcalf D. (1971). Proliferation of macrophage and granulocyte precursors in response to primary and transplanted tumors.. Isr J Med Sci.

[OCR_00769] LAPPAT E. J., CAWEIN M. (1964). A STUDY OF THE LEUKEMOID RESPONSE TO TRANSPLANTABLE A-280 TUMOR IN MICE.. Cancer Res.

[OCR_00784] Thomson D. M., Eccles S., Alexander P. (1973). Antibodies and soluble tumour-specific antigens in blood and lymph of rats with chemically induced sarcomata.. Br J Cancer.

[OCR_00791] Volkman A., Collins F. M. (1974). The cytokinetics of monocytosis in acute salmonella infection in the rat.. J Exp Med.

[OCR_00796] Whitelaw D. M. (1966). The intravascular lifespan of monocytes.. Blood.

[OCR_00800] Wrathmell A. B. (1976). The growth patterns of two transplantable acute leukaemias of spontaneous origin in rats.. Br J Cancer.

